# Studies on the Oxidation of Aromatic Amines Catalyzed by *Trametes versicolor* Laccase

**DOI:** 10.3390/ijms24043524

**Published:** 2023-02-09

**Authors:** Ivan Bassanini, Simone Grosso, Chiara Tognoli, Giovanni Fronza, Sergio Riva

**Affiliations:** 1 Istituto di Scienze e Tecnologie Chimiche-SCITEC, Consiglio Nazionale delle Ricerche, Via Mario Bianco 9, 20131 Milan, Italy; 2 Dipartimento di Scienze Farmaceutiche, Università degli Studi di Milano, Via Mangiagalli 25, 20133 Milan, Italy; 3 Istituto di Scienze e Tecnologie Chimiche-SCITEC, Consiglio Nazionale delle Ricerche, Via Luigi Mancinelli 7, 20131 Milan, Italy

**Keywords:** biocatalyzed [2 + 2] olefin cycloaddition, *T. versicolor* laccases, anilines, bio-oxidation, radical chemistry

## Abstract

The bio-oxidation of a series of aromatic amines catalyzed by *T. versicolor* laccase has been investigated exploiting either commercially available nitrogenous substrates [(*E*)-4-vinyl aniline and diphenyl amine] or ad hoc synthetized ones [(*E*)-4-styrylaniline, (*E*)-4-(prop-1-en-1-yl)aniline and (*E*)-4-(((4-methoxyphenyl)imino)methyl)phenol]. At variance to their phenolic equivalents, the investigated aromatic amines were not converted into the expected cyclic dimeric structures under *T. versicolor* catalysis. The formation of complex oligomeric/polymeric or decomposition by-products was mainly observed, with the exception of the isolation of two interesting but unexpected chemical skeletons. Specifically, the biooxidation of diphenylamine resulted in an oxygenated quinone-like product, while, to our surprise, in the presence of *T. versicolor* laccase (*E*)-4-vinyl aniline was converted into a 1,2-substited cyclobutane ring. To the best of our knowledge, this is the first example of an enzymatically triggered [2 + 2] olefin cycloaddition. Possible reaction mechanisms to explain the formation of these products are also reported.

## 1. Introduction

Laccases, blue copper oxidases [1], are enzymes whose natural function(s) are related to their ability to catalyze polymerization and depolymerization processes. As examples, fungal laccases are involved in lignin degradation, while in plants they are key players in lignification processes and cell wall formation [2].

Generally speaking, laccases are regarded as “green tools” enabling the employment of biocatalyzed processes in different fields of industrial interest [3,4]. While laccases’ main (bio)technological applications are usually found in the textile, pulp, paper, and food industries [1,3,5], according also to the large number of patents filed during the last years [6], the versatility and the surprisingly wide substrate scope of these enzymes makes them appealing for the synthesis of fine chemical and for the development of novel and green organic transformations, as we recently reviewed [7].

Radical chemistry is a trivial synthetic tool that, under the proper choice of reactants and reaction media engineering, allows to obtain complex molecular skeletons in simple one-pot processes. In this context, laccase–catalysis represents a convenient activation protocol of a normally inert Csp^2^-H bond, requiring only molecular oxygen and ad hoc designed aromatic substrates. Specifically, the laccase-mediated generation of reactive radical intermediates can be efficiently exploited to build domino, cascade, and/or one-pot ring closure processes to be applied to the preparation of heterocyclic compounds. It is noteworthy that no toxic, hazardous, and expensive metal-based chemical catalysts are needed to perform these biocatalytic processes.

**Scheme 1:**
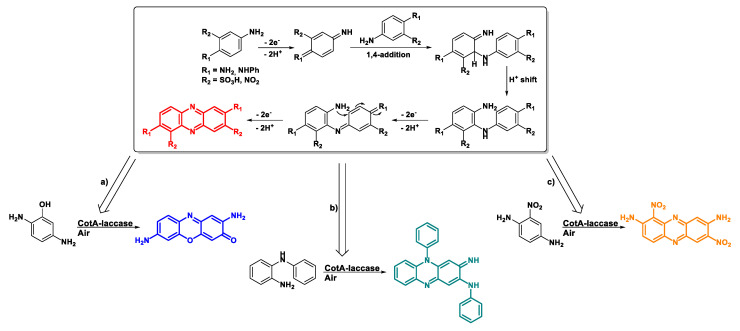
Mechanistic insights on phenazines formation via radical-cationic oxidation of substituted phenoxazinones (**a**) and phenazines (**b**,**c**) catalyzed by CotA-laccase.

Multi-step sequences composed by, e.g., a series of (pseudo)quinones formations, nucleophilic aromatic substitutions, and C-C and C-N radical couplings can be merged to synthesize nitrogenous heterocycles starting from aromatic amines. It has been shown that these transformations result in formal oxidative homo- and/or hetero-couplings involving two molecules of the same substrate or of two different partners [8]. Scheme 1 summarizes some of the results obtained by Sousa et al. in the biocatalytic oxidation of differently substituted anilines using the bacterial CotA-laccase (spore coat protein A, CotA) from *Bacillus subtilis* as the biocatalyst for the facile synthesis of phenazines and phenoxazinones [9,10,11]. These elegant biocatalytic syntheses could represent a convenient entry to analogues of bioactive phenazines [12,13,14].

Among the different species of laccases, the enzyme from *T. versicolor*, a common polypore mushroom, has been widely exploited and characterized as a biocatalyst for the development of synthetic processes based on radical chemistry and differently substituted natural or ad hoc designed phenol substrates [7,15,16]. As for most of these enzymes, the catalytic cycle of *T. versicolor* laccase involves the mono-electronic oxidation of four equivalent of an organic (reducing) substrate forming radicals at the expense of molecular oxygen, which is eventually reduced to two molecules of water. The core of the catalytic machinery is represented by a four-membered copper cluster, which is the site of oxygen coordination and reduction, water formation and release, as well as of substrate oxidation [1,3,17].

Besides being largely exploited in the (bio)technological applications mentioned above, *T. versicolor* laccase has been extensively employed in the development of green and convenient processes to afford oxygen-containing heterocyclic compounds starting from the formal oxidative homocoupling of differently substituted phenols or ad hoc designed phenolic synthetic derivatives [18,19,20]. These biocatalyzed multi-step, one-pot ring closing reactions are usually guided by the structural features of the reacted substrates that can control the profile of the molecular skeletons obtained. When novel stereocenters are formed, no control of their absolute configuration is achieved, while steric hindrance and thermodynamics drive their relative configuration.

**Scheme 2:**
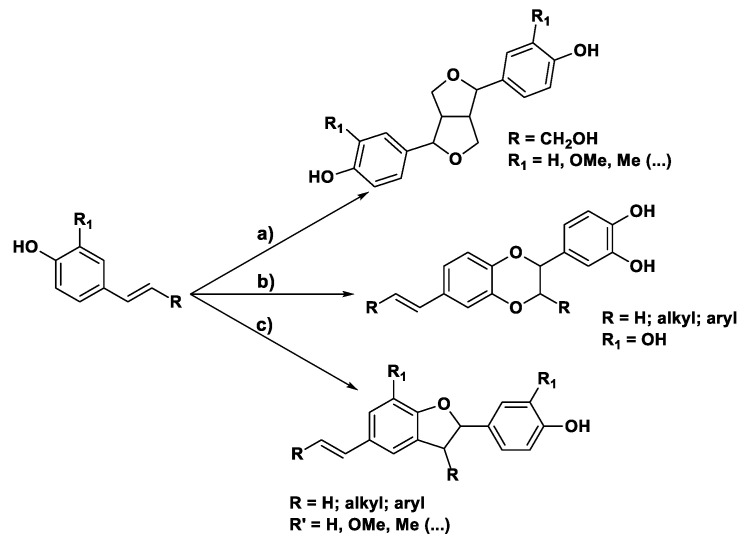
Oxygenated heterocycles synthetized via *T. versicolor* laccase catalysis: hexahydrofuro [3,4-c]furan (**a**), benzodioxane (**b**), and 2,3-dihydrobenzofuran (**c**) cores.

Specifically, when vinyl phenols and stilbenoids, molecules structurally related to the laccases’ natural substrates mono-lignols, are reacted in the presence of *T. versicolor* laccase, three different groups of oxygenated heterocycles can generally be obtained, as *trans*-racemate, as described in Scheme 2: in the presence of an allylic alcohol (R = OH) and of an alkyl substituent such as R’ bicyclic hexahydrofuro [3,4-c]furans, the core of the natural product pinoresinol [21,22,23,24], are preferentially formed (a) while benzodioxanes (b) and 2,3-dihydrobenzofurans (2,3-DHBs) (c) are obtained starting from vinyl catechols and phenols, respectively.

**Figure 1:**
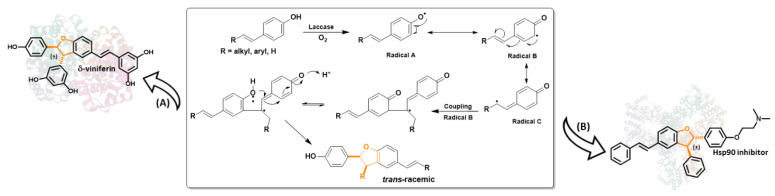
*T. versicolor* laccase-mediated domino process to 2,3-DHBs via a formal oxidative homocoupling. Examples of preparations of bioactive compounds: (**A**) δ-viniferin, a natural antioxidant able to bind to human hemoglobin [34] and (**B**) synthetic inhibitor of Hsp90 variants potentially endowed with antiproliferative activity [31].

As we extensively reported [25,26,27,28,29,30,31,32,33], 2,3-DHBs are obtained as the main products when the designed substrate (Scheme 2) is characterized by an R_1_ ‘spectator group’ (i.e., hydrogen, alkyl chains, substituted phenols) and by an R substituent that is either an alkyl or an aryl group. From a mechanistic point of view (Figure 1), the ring closure occurs via a sequence of phenol oxidation, C-C/O radical coupling, and 1,4-conjugate addition forming two novel stereocenters. In particular, we exploited *T. versicolor* laccases for the convenient chemo-enzymatic preparation of libraries of 2,3-DHB-based bioactive compounds or for the one-pot, selective manipulation of valuable natural compounds [26,31,34].

**Scheme 3:**
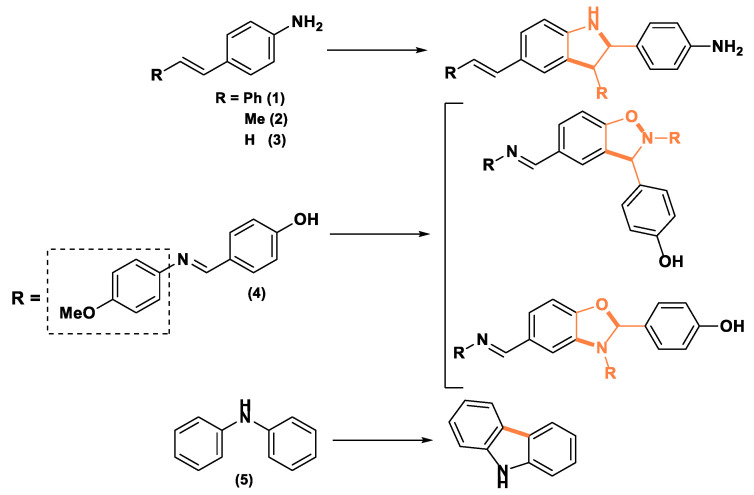
Substrates **1–5** and their likely expected laccase-catalyzed oxidation products.

As we previously discussed, while the use of laccases of different origins has been reported for the synthesis of phenazine-like compounds or generic organic dyes possessing the structure of Bandrowski’s base-like trimers (Scheme 1), [9,10,11,35] to the best of our knowledge, laccase-catalysis has not been exploited yet for oxidation of the corresponding vinyl or styryl anilines. Thus, we decided that it was worthy investigating whether *T. versicolor* laccase catalysis could be exploited to oxidize aromatic amines or imines to build indoline, oxazole/isoxazole, or carbazole skeletons, as described in Scheme 3, using the model substates **1–5**, respectively. In the following, we report and discuss the obtained results.

## 2. Results

### 2.1. Synthesis of Substrates

**Scheme 4:**
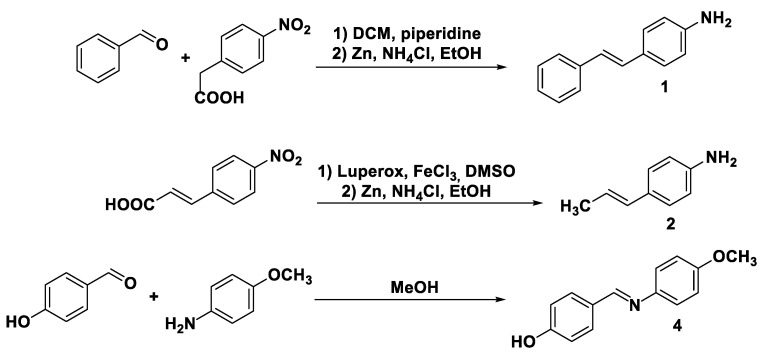
Synthetic entries to substrates **1**, **2** and **4**.

Substrates **1**, **2,** and **4** are not commercially available and therefore they were chemically synthetized for the purpose of this study (Scheme 4).

The styryl aniline **1** was obtained via a sequence of Knoevenagel condensation, involving *p*-nitro benzoic acid and benzaldehyde working in the presence of piperidine as a base, and the reduction of this nitro group mediated by metallic zinc.

The *E*-configured methyl vinyl aniline **2** was similarly obtained from the Zn-promoted reduction of the corresponding nitro-derivative which, in turn, was prepared from the decarboxylation of the commercially available (*E*)-3-(4-nitrophenyl)acrylic acid in the presence of FeCl_3_.

Finally, imine **3** was easily afforded by the condensation of *p*-hydroxy benzaldehyde and *p*-methoxy aniline in pure MeOH at room temperature.

### 2.2. Laccases-Mediated Biooxidations

#### 2.2.1. Substrate **1**, **2,** and **4**

The biocatalyzed oxidations of aryl and alkyl substituted vinyl anilines **1** and **2** and of imine **4** was explored via a series of small-scale reactions monitored by TLC analysis.

Standard oxidation conditions for *T. versicolor* laccase catalysis (acid pH, 27 °C) were applied along with modified protocols in which the pH was moved up to neutral (7.0) and slightly basic (up to 8.0) values. In all the cases (data not shown), starting materials and/or a complex mixture of by-products were isolated.

**Figure 2:**
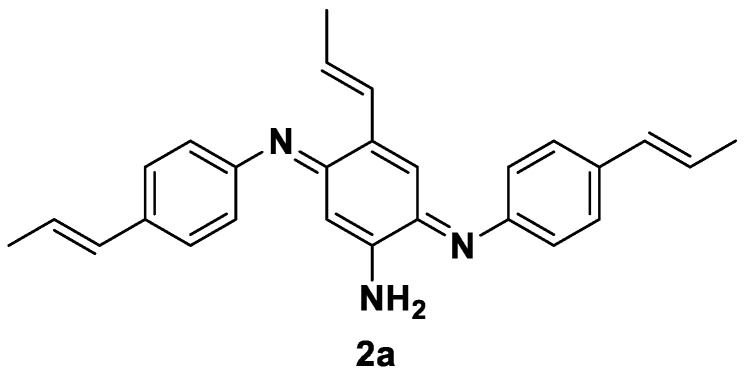
Hypothesized structure of the trimeric Bandrowski’s base-like product **2a** formed in traces from **2** [36].

Only in the case of substrate **2**, traces of a trimeric structure already reported for the laccase mediated bio-oxidation of similar aryl amines [35,36] were detected via mass spectroscopy, but it was not possible to properly isolated it. According to the literature references, the structure reported in Figure 2 might be hypothesized.

In conclusion, to our disappointment, no evidence of discrete dimeric products could be observed with these three substrates.

#### 2.2.2. Substrate **3**

The bio-oxidation of compound **3** was at first investigated by selecting the proper reaction medium and then optimized in terms of enzyme loading and general parameters as reaction time or temperature. Control reactions were also performed to exclude the presence of spontaneous oxidation in the investigated reaction media.

The optimized conditions for this biotransformation were identified as adding portion wise *T*. *versicolor* laccase, 36 U per mmol of substrate, to an acetate buffer solution of vinyl aniline.

**Scheme 5:**
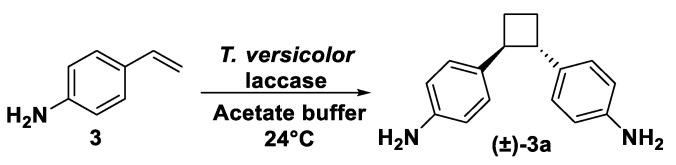
Reaction of vinyl aniline **3** to give **3a** via *T. versicolor* laccase–catalysis.

The reaction, shaken at 24 °C, was followed using TLC attesting the disappearing of the starting material and the formation of a main, more polar compound (R_f_ = 0.25 in petroleum ether–ethyl acetate = 7:3), which was purified by flash column chromatography on silica gel (FC). Product **3a** was then characterized by means of NMR spectroscopy and mass spectrometry and was identified as a dimeric structure corresponding to a 4,4′-(cyclobutane-1,2-diyl)dianiline skeleton obtained as a putative *trans*-isomer (as a racemate) in 40% of isolated yield (**3a**, Scheme 5).

#### 2.2.3. Substrate **5**

As described for **3**, a small-preparative scale bio-oxidation of substrate **5** was conducted after a short investigation of the proper reaction medium and enzyme loading.

**Scheme 6:**
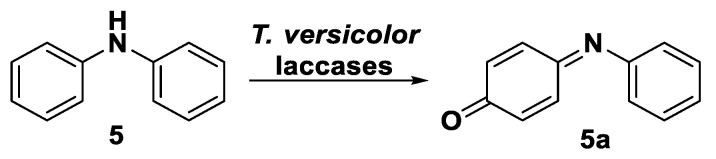
Reaction of diphenyl amine **5** to give **5a** via *T. versicolor* laccase–catalysis.

Specifically, **5** was reacted with *T. versicolor* laccase in a 30% *v*/*v* solution of dioxane in acetate buffer in the presence of 1.5 U mL^−1^ of biocatalyst. The reaction was incubated for 24 h at 27 °C and followed by TLC analysis until complete disappearance of the starting material. A more polar compound (R_f_ = 0.34 in petroleum ether–ethyl acetate = 9:1) was formed as a main product along with a complex mixture of apparently indistinguishable spots. The main product was isolated by means of FC and fully characterized via NMR spectroscopy and mass spectrometry. Based on these analyses, the structure of the obtained chemical species (**5a**, Scheme 6) could be assigned to a 4-(phenylimino)cyclohexa-2,5-dien-1-one skeleton obtained in a 10% of isolated yield.

## 3. Discussion

As it has been described in the introduction, the CotA-laccase-catalyzed dimerization of substituted anilines to give phenazines is a well-assessed methodology. Moreover, the enzymatic oligomerization and polymerization of anilines is well-documented in the literature, as it has been recently reviewed [36]. However, the structural characterization of dimers and trimers formed during the early stages of the biocatalyzed oxidative polymerization of these compounds is missing in most cases.

In an attempt to tackle this problem, at least with specific aniline derivatives, this research was focused on the π-conjugated vinyl anilines **1–3**, with the aim of verifying whether these aromatic amines behaved like their oxygenated cognates, forming indole skeletons under laccase oxidation [25,26,27,28,29,30,31,32,33]. Moreover, we also considered compounds **4** (an easily synthesized imine) and the aromatic secondary amine **5** as possible precursors of cyclic nitrogen-containing heterocycles, respectively of oxazole/isoxazole or carbazole skeletons (Scheme 3). Unfortunately, our approach failed to give the hypothesized results. However, in two cases it was possible to isolate products with defined and unexpected chemical structures.

As described in the previous paragraph, the bio-oxidation of **2** gave only traces of the trimeric compound **2a**, previously reported in the literature [36], as attested by the mass spectrum reported in Figure S1 (M* = 293.2 Da).

**Scheme 7:**
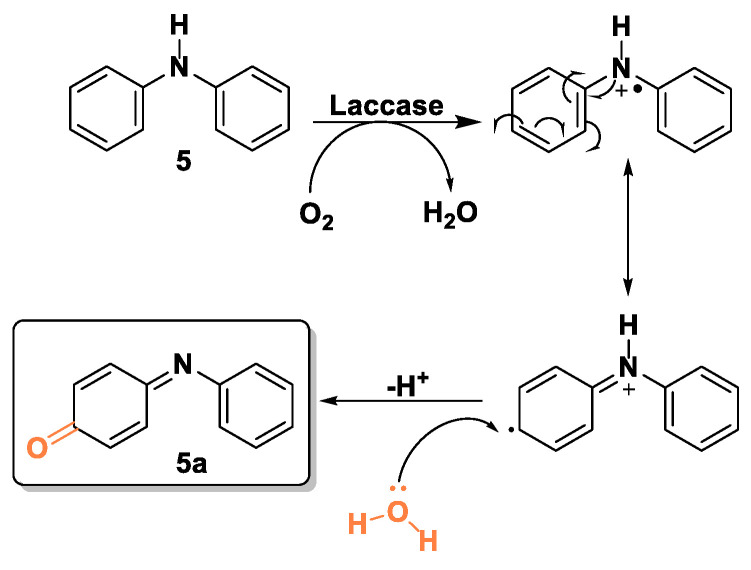
Proposed mechanism for the biooxidation of **5**.

The elucidation of the structure of compound **5a**, obtained by the laccase-catalyzed oxidation of diphenyl amine **5**, was not straightforward. The mass spectrum (Figure S2), with a molecular value of 184.1 Da, suggested the presence of an oxygenated substituent. A careful inspection of the signals of the ^1^H NMR spectrum and the exploitation of bidimensional techniques (Figure S2) allowed to identify the structure **5a**, formed according to the following hypothesized mechanism (Scheme 7).

Even more puzzling was the characterization of the main product obtained by the laccase-catalyzed oxidation of 4-vinyl aniline (**3**), isolated in 40% yield. Its exact molecular mass at 239.15410 Da was compatible with a dimeric structure with a brute formula C_16_H_10_N_2_. Its ^1^H NMR spectrum, far from being trivial (Figure S3), was deeply investigated and, besides aromatic systems, no signs of the original Csp^2^-H olefinic protons could be identified. In place of those signals, a multiplet centered at 3.36 ppm could be identified. Given the nature of the reacted substrate, the molecular mass of the product obtained, and the identification of novel signals in the region of Csp^3^-H protons, we hypothesized that **3a** could be a disubstituted cyclobutane skeleton. To identify the obtained regioisomer, we carefully looked in the literature for reports of ^1^H-NMR characterization of 1,2 and 1,3-substituted cyclobutane rings. The ^1^H spin system characterizing this family of cyclobutane rings can be described as AA’BB’XX’, where X and X’ are the isochronous methine nuclei. In these systems, the width at half height of the signal of the XX’ nuclei depends essentially on the sum of the vicinal coupling constants (J), e.g., J(AX) and J(BX) for X, since the J across the four bonds are small (about 1 Hz) in four membered rings. In cyclobutanes, vicinal J are usually found between 7 and 10 Hz. Thus, the width of the XX’ signal of a 1,2-disubstituted cyclobutane should measure 15–20 Hz (only one adjacent methylene) while 1,3-disubstituted cyclobutanes could reach values greater than 30 Hz (two adjacent methylenes). These considerations allowed us to identify **3a** as a 1,2-disubstituted cyclobutane as the width of the signal at 3.36 ppm (corresponding to XX’ nuclei) was found to be 17.0 Hz, in perfect agreement with that reported by Raza et al. in the ^1^H NMR characterization of diphenyl cyclobutanes [37]. As far as the relative stereochemistry of **3a** concerns, we hypothesize a *trans*-configured cyclobutane due to the similarity of the fine structure of the NMR spectrum with that of the 1,2-*trans*-diphenylcyclobutane [37] and the fact that generally laccases-mediated dimerization reactions are driven by thermodynamic factors which generally lead to the formation of the more stable and less hindered *trans*-systems [7].

**Scheme 8:**
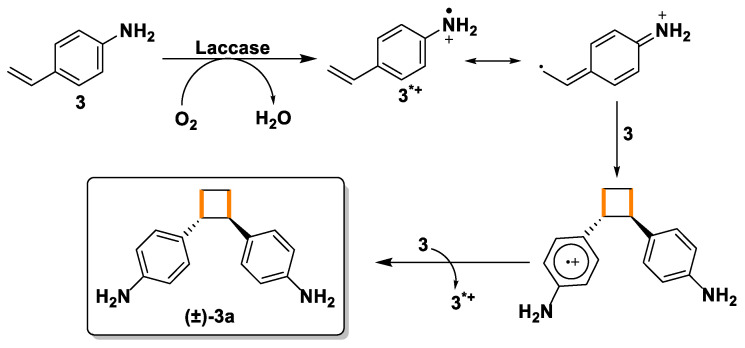
Proposed mechanism for the bio-oxidation of **3**: a laccase-initiated [2 + 2] cycloaddition via a radical-cation activated species [39,40,41].

This structure was quite an unexpected molecular skeleton to be formed under laccase catalysis. As a possible rationale, we propose the following mechanism (Scheme 8) for the formation of **3a,** which relies on the well-documented [2 + 2] photochemical cycloadditions of olefins [38].

The isolation of **3a** represents, to the best our knowledge, the first example of a biocatalytic [2 + 2] cycloaddition of olefins. In this reaction, *T. versicolor* laccase is expected to act as an initiator by activating the fully conjugated π-system of vinyl aniline **3** to the radical cation 3*^+^, an intermediate reported as pivotal in this kind of cyclization reactions [39,40,41].

Interestingly, the same cyclobutane dimeric structures were not isolated with the vinyl anilines **1** and **2**. It could be possible to argue that steric hindrance or ring-tension by the additional presence of phenyl and methyl substituents in the position C-2 and C-4 could have prevented the cycloaddition reaction. However, several reports can be found in the literature dealing with the preparation of tri- and tetra-substituted cyclobutanes exploiting protocols of metal-catalyzed [2 + 2] olefin photocycloadditions [38,42,43,44]. For all these reasons, a detailed investigation of this laccase-mediated process will be conducted by us, using different fully conjugated π-systems as substrates.

## 4. Materials and Methods

All reagents were of the highest purity grade from commercial suppliers: Merck (St Louis, MO, USA) or VWR (Radnor, PA, USA).

Laccase from *Trametes versicolor* was from Sigma-Aldrich. The enzyme was used based on their respective activities evaluated according to literature assay based on the ABTS (2,2′-azino-bis(3-ethylbenzothiazoline-6-sulfonic acid)) as model substrate. [32]

Biotransformations were performed in a G24 Environmental Incubator New Brunswick Scientific Shaker (Edison, USA) or in a Thermomixer Comfort (Eppendorf, DE).

Reactions were monitored by thin-layer chromatography (TLC) (precoated silica gel 60 F254 plates (Merck, DE)); development with UV lamp, Komarovsky reagent (1 mL 50% ethanolic H_2_SO_4_ with 10 mL 2% methanolic 4-hydroxybenzaldehyde), a 20% solution of H_2_SO_4_ in ethanol or a molybdate reagent ((NH_4_)_6_Mo_7_O_24_·_4_H_2_O, 42 g; Ce(SO_4_)_2_, 2 g; H_2_SO_4_ conc., 62 mL; made up to 1 L of deionized water). Flash chromatography: silica gel 60 (70–230 mesh, Merck, DE).

NMR spectra were recorded with a Bruker AC spectrometer (400 or 500 MHz) in [D_4_]MeOH, [D_6_]DMSO or [D_1_]CHCl_3_. Mass spectra were recorded with a Bruker Esquire 3000 Plus spectrometer.

High-resolution mass spectra (HRMS) were conducted on FT-Orbitrap mass spectrometer in positive electrospray ionization (ESI).

### 4.1. Synthesis of Dubstrates

#### 4.1.1. (*E*)-1-nitro-4-styrylbenzene

As we previously reported for the synthesis of this compound [31], a solution of benzaldehyde (100 mg, 0.9 mmol, 1 eq) and 4-nitrophenylacetic acid (0.51 g, 2.8 mmol, 3 eq) was prepared with 15 mL of CH_2_Cl_2_ at r.t. Piperidine (280 mL, 2.8 mmol, 3 eq) was added and the resulting mixture was gradually heated to 130 °C, distilling the solvent. The resulting neat mixture was left reacting at 130 °C for 24–48 h. After that, the crude residue was analyzed by TLC (CHCl_3_-acetone = 95:5) and purified by flash column chromatography (petroleum ether-EtOAc = 96:4), obtaining the desired (*E*)-stilbene as a yellow solid (190 mg, 89% yield). ^1^H-NMR (400 MHz; [D_4_]MeOH, r.t.): δ 8.22–8.19 (AA’BB’ system, 2H), 7.74–7.71 (AA’BB’ system, 2H), 7.49–7.47 (AA’BB’ system, 2H), 7.34 (d, J 16.4 Hz, 1H), 7.10 (d, J 16.4 Hz, 1H), 6.84–6.81 (AA’BB’ system, 2H); ^13^C-NMR (101 MHz; [D_4_]MeOH, r.t.): δ 146.9, 143–9, 136.3, 133.4, 129.0, 129.9, 127.1, 126.9, 126.4, 124.2. MS, *m*/*z* ESI = 264.0 [M + Na]^+^.

#### 4.1.2. (*E*)-4-styrylaniline (Substrate **1**)

Zinc power (72 mg, 1.1 mmol, 5 eq) and ammonium chloride (60 mg, 1.1 mmol, 5 eq) were added to a stirred solution of *p*-nitrostilbene (50 mg, 0.2 mmol, 1 eq) in EtOH (0.52 mL). The resulting reaction mixture was heated to 90 °C for 6 h, monitored by TLC (petroleum ether–EtOAc = 9: 1). The reaction mixture was cooled to r.t., filtered through a celite^®^ pad, and volatiles were evaporated under reduced pressure. The crude residue was diluted with EtOAc (20 mL) and washed with a solution of NaHCO_3_ (3 × 10 mL) and brine (3 × 10 mL), dried over Na_2_SO_4_ and the solvent evaporated under reduced pressure to afford the desired product as a white foam (32 mg, 90% yield). ^1^H-NMR (400 MHz; [D_4_]MeOH, r.t.): δ 7.48 (AA’BB’ system, 2H), 7.22 (t, J 7.4 Hz, 1H), 7.03 (d, J 16.3, 1H), 6.93 d, J 16.3, 1H), 6.68 (d, J 8.5, 1H), 3.74 (brs, 2H). ^13^C-NMR (101 MHz; [D_4_]MeOH, r.t.): δ 146.3, 138.1, 129.0, 128.7, 127.0, 126.2, 125.2, 115.3. MS (ESI): calcd for [C_14_H_14_N]^+^ 196.1126, found 169.1225.

#### 4.1.3. (*E*)-1-nitro-4-(prop-1-en-1-yl)benzene

A mixture of 4-nitro cinnamic acid (290 mg, 1.5 mmol, 1 eq), DTBP (2-(tert-butylperoxy)-2-methylpropane, 550 μL, 3 mmol, 2 eq), FeCl_3_ (81 mg, 0.3 mmol, 20 mol%), and DMSO (10 mL) was stirred in a round-bottom flask under nitrogen atmosphere at 130 °C overnight. After that, the mixture was poured into EtOAc (10 mL) and washed with water (25 mL). The aqueous phase was extracted with EtOAc (15 mL). The combined organic layers were dried over Na_2_SO_4_ and the solvent evaporated under reduced pressure. The crude product was purified by purified by flash column chromatography (petroleum ether-EtOAc = 97:3), obtaining the desired compound as a sightly yellow solid (43 mg, 17% yield). ^1^H-NMR (400 MHz; [D_4_]MeOH, r.t.): δ 8.14 (AA’BB’ system, 2H), 7.43 (AA’BB’ system, 2H), 6.46 (s, 2H), 1.94 (d, J 4.9 Hz, 3H). ^13^C-NMR (101 MHz; [D_4_]MeOH, r.t.): δ 146.5, 144.5, 131.4, 129.6, 126.3, 124.0, 18.84. MS, *m*/*z* ESI = 186.0 [M + Na]^+^.

#### 4.1.4. (*E*)-4-(prop-1-en-1-yl)aniline (Substrate **2**)

Following the same reduction protocol applied for the preparation of (**1**), substrate **2** was obtained as a white foam (93% isolated yield, 80 mg) starting from 106 mg of (E)-1-nitro-4-prop-l-en-1-yl)benzene. ^1^H-NMR (400 MHz; [D_4_]MeOH, r.t.): δ 7.14 (AA’BB’ system, 2H), 6.62 (AA’BB’ system, 2H), 6.62 (d, J 8.5, 1H), 6.30 (dd, J 15.7, 1.4 Hz, 1H), 6.30 (dq, J 15.7, 6.6 Hz, 1H), 3.60 (bs, 1H), 1.84 (dd, J 6.6 Hz, 1.4 Hz, 3H). ^13^C-NMR (101 MHz; [D_4_]MeOH, r.t.): δ 145.3, 130.8, 128.9, 126.9, 122.0, 115.3, 18.4. MS (ESI): calcd for [C_9_H_12_N]^+^ 133.0891, found 133.0892.

#### 4.1.5. (*E*)-4-(((4-methoxyphenyl)imino)methyl)phenol (Substrate **4**)

p-Hydroxy benzaldehyde (100 mg, 0.8 mmol, 1 eq) and p-methoxy aniline (101 mg, 0.8 mmol, 1 eq) were dissolved in pure EtOH (4.1 mL). The obtained solution was stirred under reflux for 3 h observing the formation of a yellow precipitate, which was isolated by filtration and crystallized from pure EtOH affording the desired imine (80% yield). ^1^H-NMR (400 MHz; [D_6_]DMSO, r.t.): δ 10.03 (s, 1H), 8.47 (s, 1H), 7.75 (AA’BB’ system, 2H), 7.33–7.11 (AA’BB’ system, 2H), 7.04–6.89 (AA’BB’ system, 2H), 6.87 (AA’BB’ system, 2H), 3.77 (s, 3H). ^13^C-NMR (101 MHz; [D_4_]MeOH, r.t.): δ 160.8, 160.0, 159.0, 144.4, 131.2, 130.7, 129.8, 122.4, 116.2, 116.1, 116.0, 115.6, 55.8. MS (ESI): calcd for [C_14_H_13_NO**_2_** + 1]^+^ 278.0946, found 278.0944.

### 4.2. Biooxidations with T. versicolor Laccase

#### 4.2.1. Oxidation of Vinyl Aniline (Substate **3**)

Vinyl aniline (30 mg, 0.3 mmol) was dissolved in 1.5 mL of sodium acetate buffer (pH 5, 50 mM) and incubated with *T. versicolor* laccase (36 U mmol^−1^_substrate_) overnight at 27 °C and 180 rpm in an orbital thermoshaker. After attesting the formation of a more polar UV-visible spot in TLC analysis (petroleum ether–EtOAc = 1:1), an additional aliquot of enzyme was added doubling its concentration and the mixture was left reacting for 6 h. The reaction mixture was then extracted with EtOAc and the combined organic layers were dried over Na_2_SO_4_ and concentrated in vacuo affording a crude mixture. The more polar spot (compound **3a**, R_f_ = 0.25 in petroleum ether–EtOAc = 7:3) was isolated by means of flash column chromatography on silica gel (petroleum ether–EtOAc = 7:3 → 4:6) and fully characterized (isolated yield = 40%). ^1^H-NMR (400 MHz; CDCl_3_, r.t.): δ 7.01 (AA’BB’ system, 2H), 6.61 (AA’BB’ system, 2H), 3.42–3.34 (m, 1H), 2.25–2.19 (m, 1H), 2.06–2.02 (m, 2H). ^13^C-NMR (101 MHz; CDCl_3_, r.t.): δ 144.3, 135.1, 127.5, 119.5, 115.1, 47.9, 25.9. MS (ESI): calcd for [C_16_H_10_N_2_]^+^ 239.15428, found 239.15410.

#### 4.2.2. Oxidation of Diphenyl Amine (Substate **5**)

Diphenyl amine (100 mg, 0.6 mmol) was dissolved in 2.5 mL of dioxane and added 5.8 mL of acetate buffer (pH 5, 50 mM) containing *T. versicolor* laccase (1.5 U mmol^−1^_substrate_) and incubated overnight at 27 °C and 180 rpm in an orbital thermoshaker. After attesting the formation of a more polar UV-visible spot in TLC analysis (petroleum ether–EtOAc = 9:1) and the disappearance of the starting material, the reaction mixture was then extracted with EtOAc, and the combined organic layers were dried over Na_2_SO_4_ and concentrated in vacuo affording a crude mixture. The polar spot (compound **5a**, R_f_ = 0.34 in petroleum ether–EtOAc = 9:1) was isolated by means of flash column chromatography on silica gel (petroleum ether–EtOAc = from 95:5 to 90:10) and fully characterized (isolated yield = 10%). ^1^H-NMR (400 MHz; CDCl_3_, r.t.): δ 7.41 (t, J 7.8 Hz, 1H), 7.31 (dd, J 10.0, 2.6 Hz, 1H), 7.27–7.22 (m, 1H), 7.09 (dd, J 10.3, 2.6 Hz, 1H); 6.89 (d, J 10.1 Hz, 1H), 6.70 (dd, J 10.1, 2.1 Hz, 1H), 6.54 (dd, J 10.3, 2.1 Hz, 1H). ^13^C-NMR (101 MHz; CDCl_3_, r.t.): δ 187.7, 157.5, 149.6, 142.0, 133.7, 133.0, 192.2, 128.4, 126.6, 120.8. MS (ESI): calcd for [C_12_H_10_NO]^+^ 184.0762, found 184.0764.

## 5. Conclusions

The laccase-catalyzed oxidation of a series of aromatic amines has been described. At variance of their phenolic equivalents, it was not possible to isolate the expected nitrogenous cyclic dimeric structures and the oxidation proceeded to give complex oligomeric and polymeric products.

Interestingly, while diphenyl amine gave an unexpected but trivial oxidized product, the laccase-mediated oxidation of vinyl aniline resulted in the isolation a 1,2-*trans*-disubstituted cyclobutane, possibly via a radical-cationic [2 + 2] olefin cycloaddition. This unexpected but highly valuable result represents the first biocatalyzed example of this reaction, which is presently a hot topic in photo and organometallic catalysis, based on the number of reports published just in the last five years. While this work concludes our long journey on the laccase-catalyzed oxidation of vinyl derivatives to give heterocyclic compounds, at the same time it opens a novel investigation on this laccase-mediated annulation reaction.

## Data Availability

Not applicable.

## Supplementary Materials

The following supporting information can be downloaded at: https://www.mdpi.com/article/10.3390/ijms24043524/s1.
