# Supplementary material for: Studies on the Oxidation of Aromatic Amines Catalyzed by Trametes versicolor Laccase
Source: Int J Mol Sci. 2023 Feb 9;24(4):3524. doi: 10.3390/ijms24043524 (PMC9963649; doi:10.3390/ijms24043524)
Supplement: Supplementary file 1 [file ijms-24-03524-s001.zip › ijms-2194368-supplementary.pdf]

# Supporting information

**INDEX**

1. Figure S1. Mass spectrum of **2a** ....................S2

2. Figure S2. Characterization of compound **5a**: 1D $^{1}H$, $^{13}C$ NMR spectra and 2D COSY....................S3

3. Figure S3. Characterization of compound **3a**: HR-MS spectrum 1D $^{1}H$, $^{13}C$ NMR spectra, 2D COSY and HSQC spectra ....................S6

## 1. Figure S1. Mass spectrum of 2a

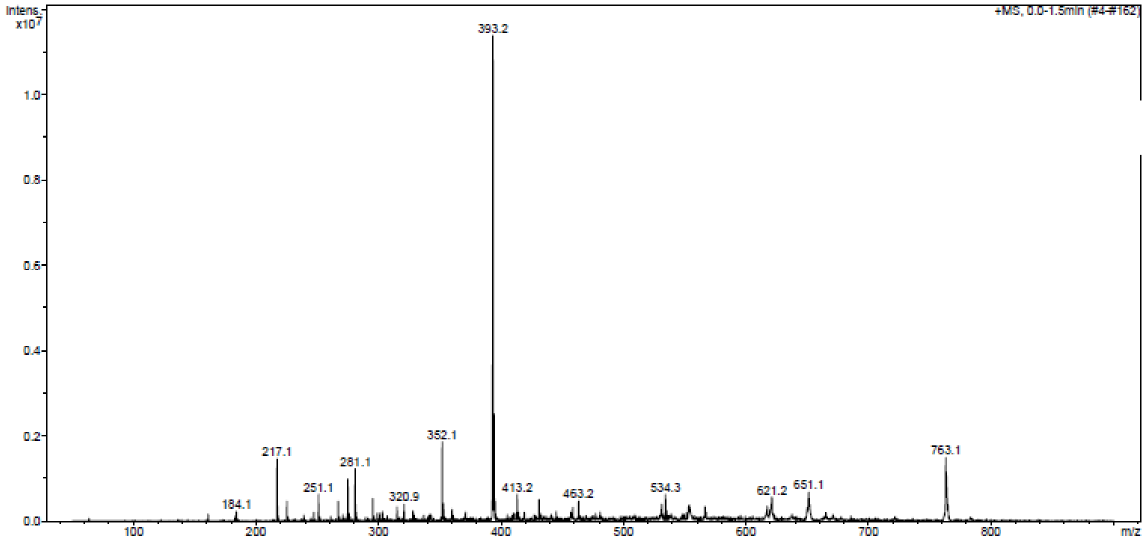


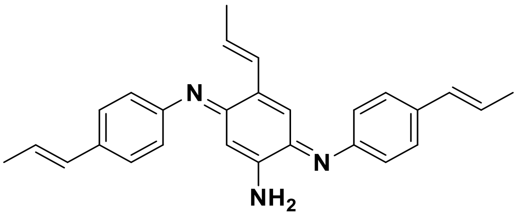


**2a, MW= 393.2**

2. **Figure S2. Characterization of compound 5a: 1D $^{1}$H, $^{13}$C NMR spectra and 2D COSY and mass spectrum**

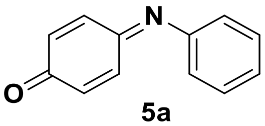

**5a**

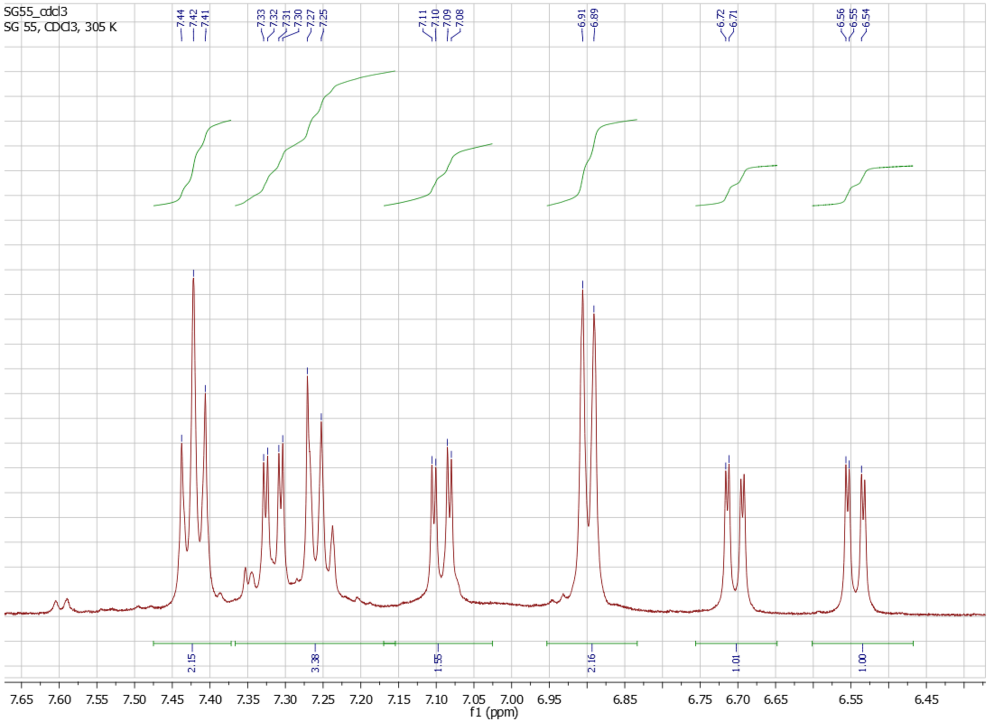

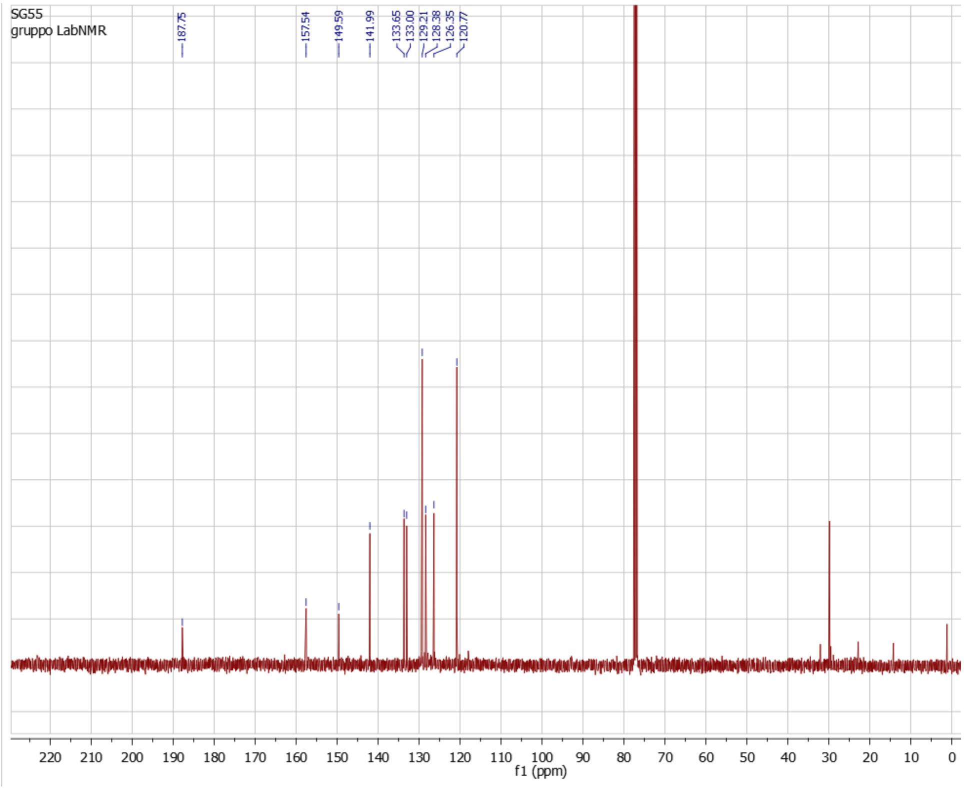

SG55
gruppo LabNMR
187.75
157.54
149.59
141.99
133.65
133.00
129.21
128.38
126.35
120.77
220 210 200 190 180 170 160 150 140 130 120 110 100 90 80 70 60 50 40 30 20 10 0
f1 (ppm)


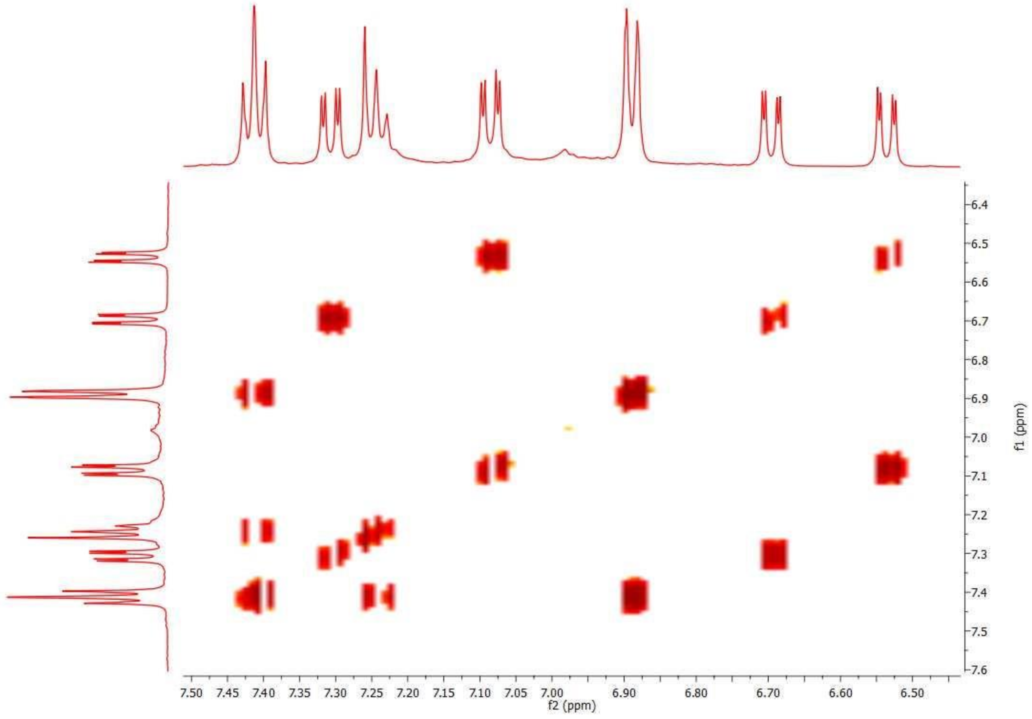

7.50 7.45 7.40 7.35 7.30 7.25 7.20 7.15 7.10 7.05 7.00 6.90 6.80 6.70 6.60 6.50
f2 (ppm)
6.4 6.5 6.6 6.7 6.8 6.9 7.0 7.1 7.2 7.3 7.4 7.5 7.6
f1 (ppm)

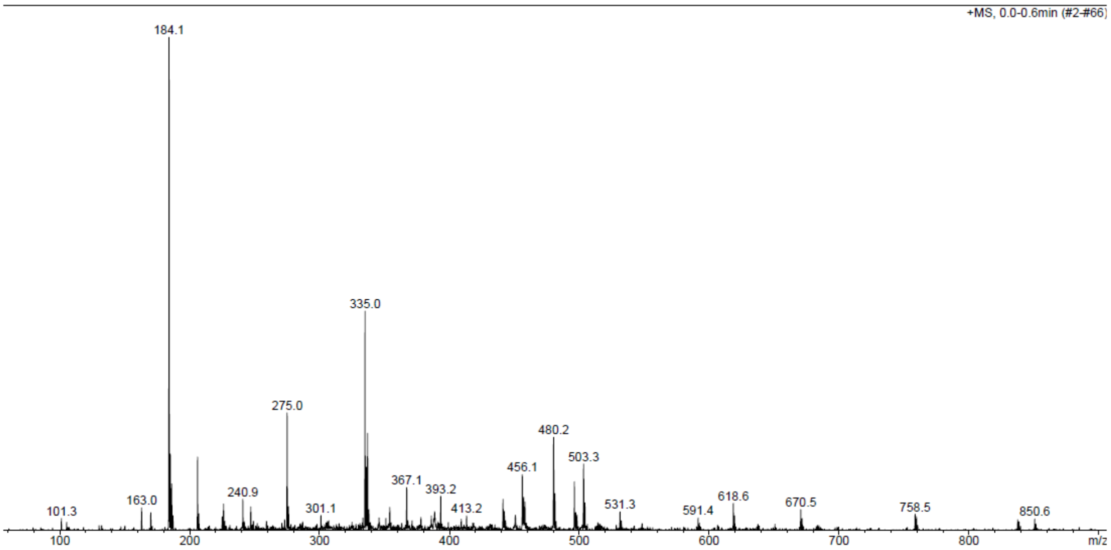
+MS, 0.0-0.6min (#2-#66)
184.1
335.0
275.0
480.2
503.3
456.1
367.1
393.2
240.9
163.0
101.3
301.1
413.2
531.3
591.4
618.6
670.5
758.5
850.6
100
200
300
400
500
600
700
800
m/z

3. **Figure S3. Characterization of compound 3a: HR-MS spectrum 1D $^{1}$H, $^{13}$C NMR spectra, 2D COSY and HSQC spectra**

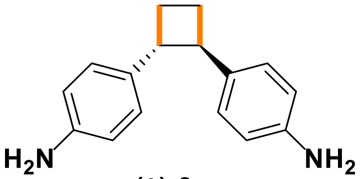

(±)-3a

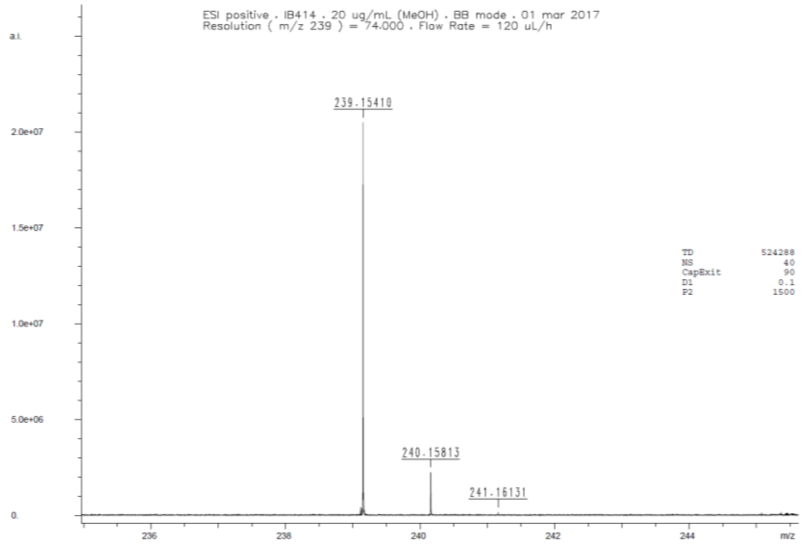


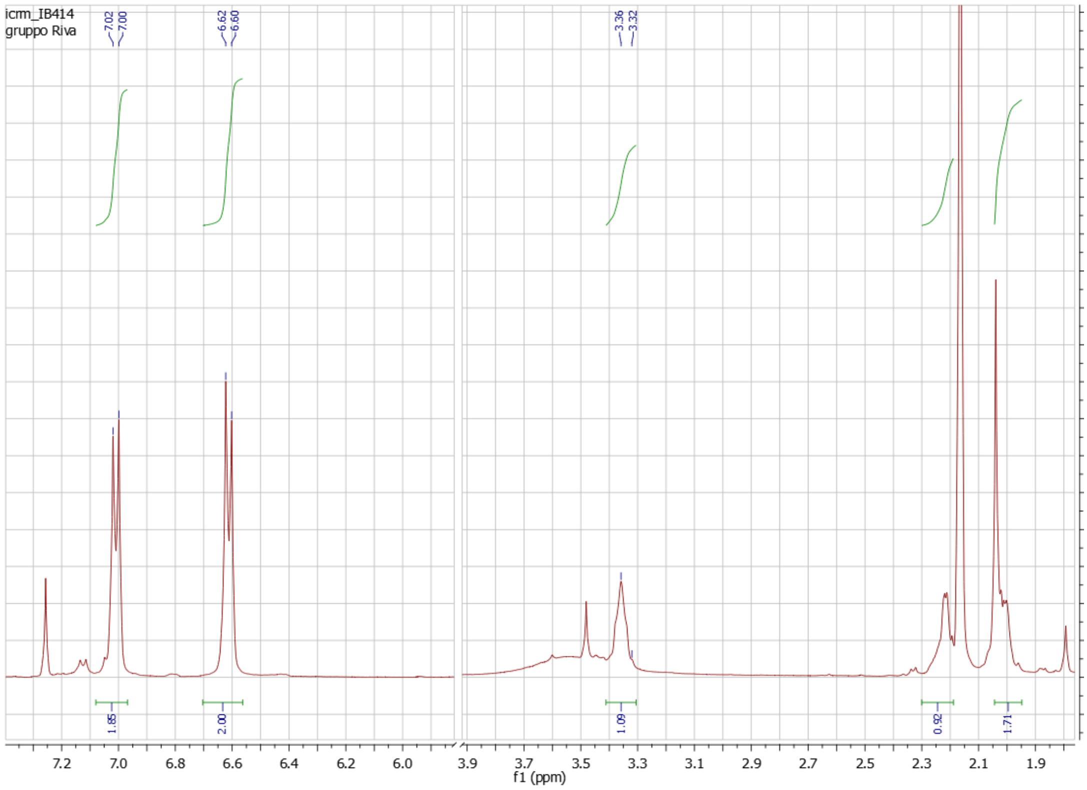

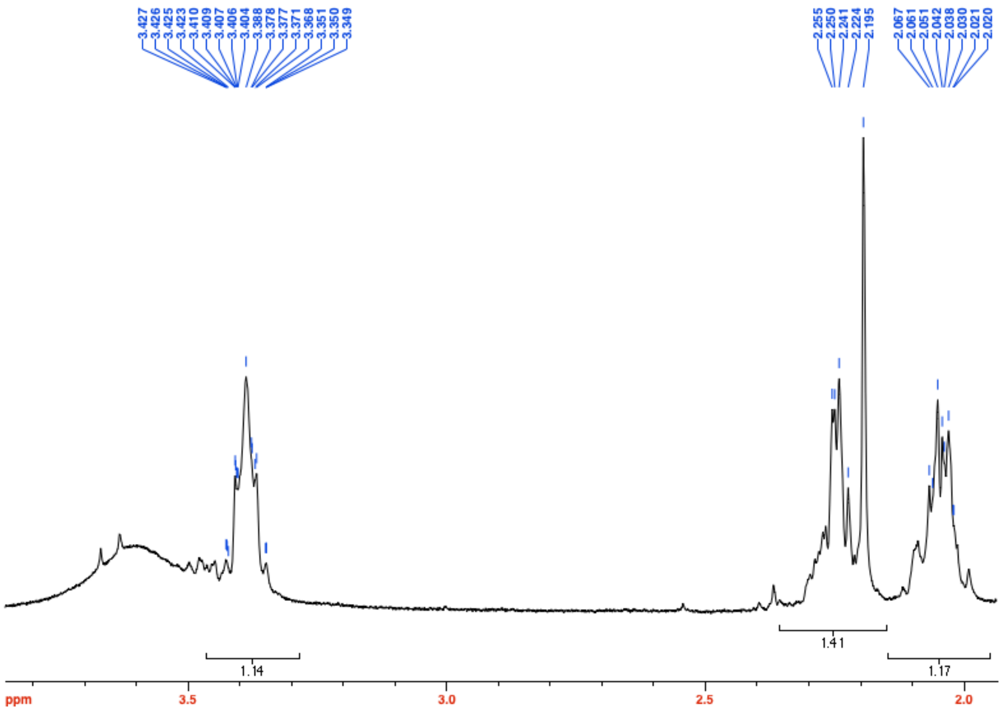
3.427
3.426
3.425
3.423
3.410
3.409
3.407
3.406
3.404
3.388
3.378
3.377
3.371
3.368
3.351
3.350
3.349
2.255
2.250
2.241
2.224
2.195
2.067
2.061
2.051
2.042
2.038
2.030
2.021
2.020
1.14
1.41
1.17
ppm
3.5
3.0
2.5
2.0

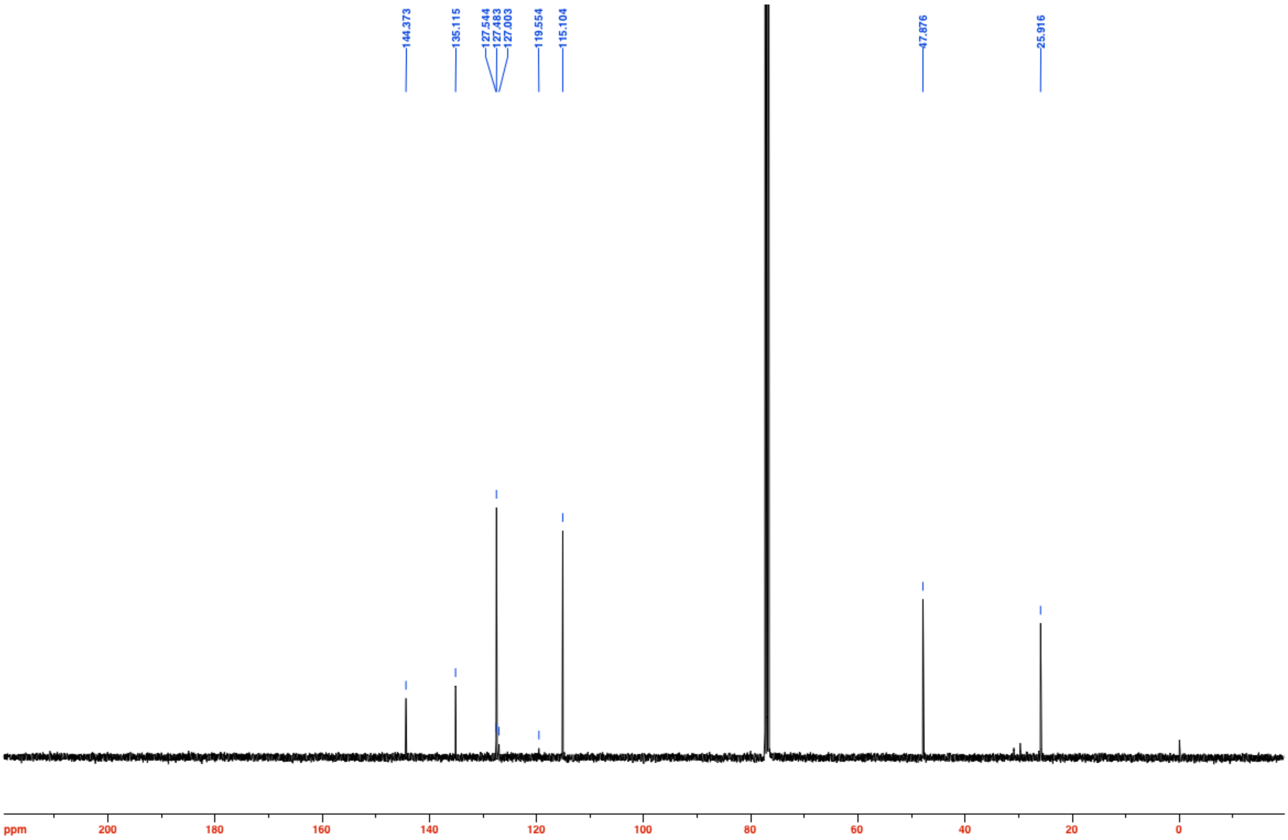
144.373
135.115
127.544
127.483
127.003
119.554
115.104
47.876
25.916
ppm
200
180
160
140
120
100
80
60
40
20
0

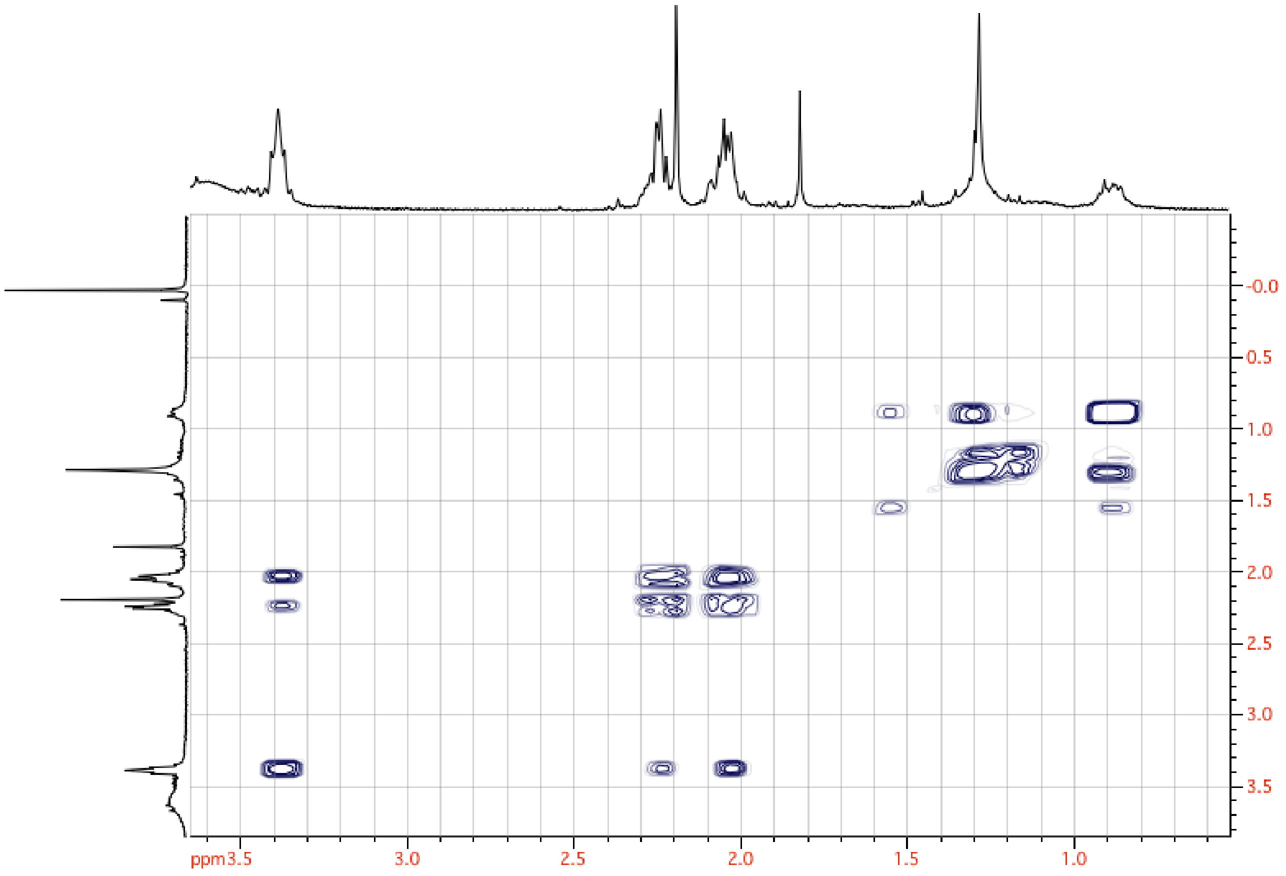
ppm
3.5
3.0
2.5
2.0
1.5
1.0
-0.0
0.5
1.0
1.5
2.0
2.5
3.0
3.5

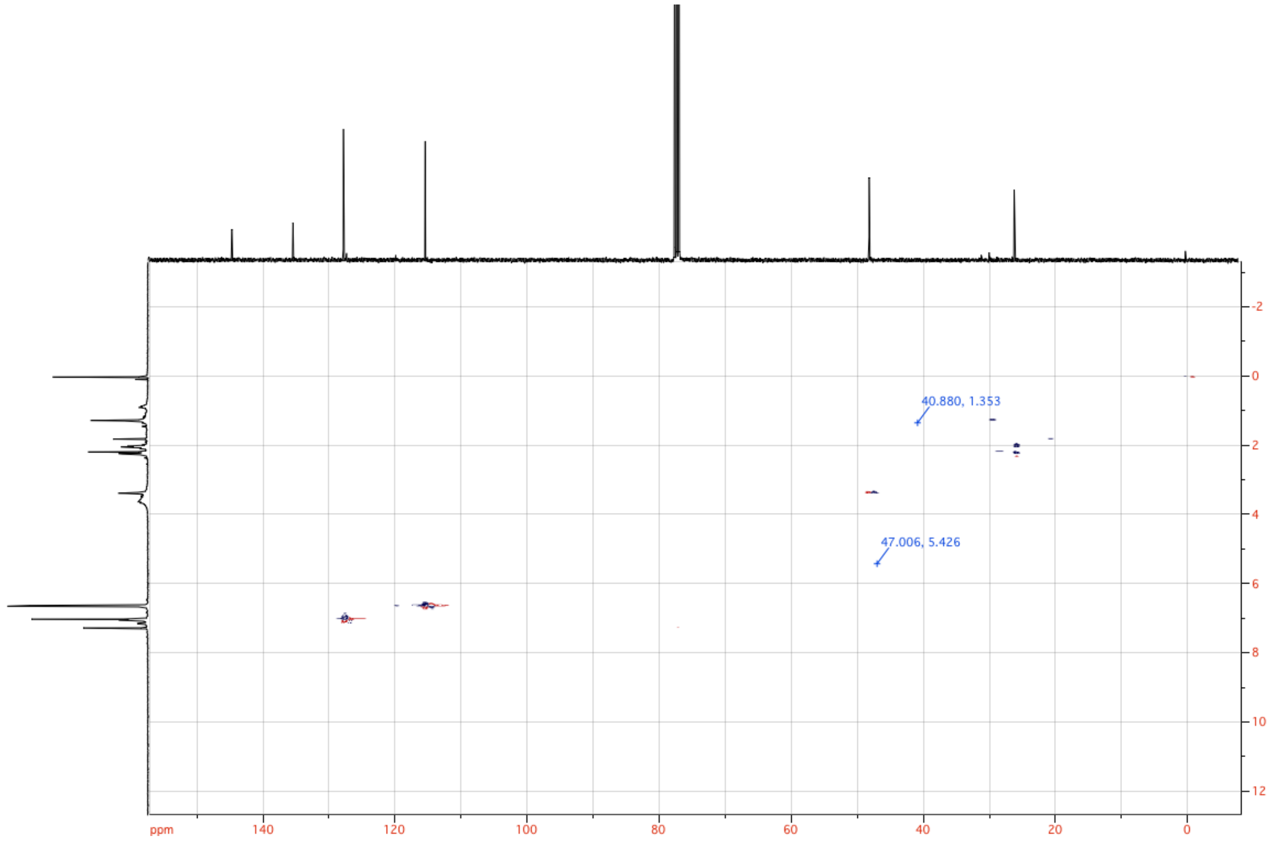
40.880, 1.353
47.006, 5.426
ppm
140
120
100
80
60
40
20
0
-2
0
2
4
6
8
10
12
